# Lipopolysaccharide (LPS) segmental lung challenge in nonhuman primates – a model of airway inflammation

**DOI:** 10.1186/1476-9255-10-S1-P29

**Published:** 2013-08-14

**Authors:** Franklin J Schlerman, Andrea G Bree, Michael D Wadanoli, Samuel J Goldman, Cara MM Williams, Joseph P Sypek

**Affiliations:** 1 Inflammation and Remodeling Research, Pfizer Cambridge Massachusetts, 02140, USA

## 

We developed a reproducible model of neutrophilic inflammation in cynomolgus monkeys (*Macaca fascicularis*) in order to study potential novel anti-inflammatory compounds intended for respiratory diseases such as COPD and asthma. Using a pediatric bronchoscope, a baseline bronchoalveolar lavage (BAL) was performed on the left lung prior to LPS segmental challenge to the right lung. At 24, 72 and 168 hours post challenge, BAL fluid (BALF) was collected to assess the level of pulmonary inflammation. At both 24 and 72 hours (Hr) post LPS challenge, total BAL cells were significantly elevated over baseline. BAL cell differentials revealed that mean percent neutrophils significantly increased from baseline (1.3±1.6%) to (74.0 ± 21.9% at 24 Hr and 26.6 ± 21.0% at 72 Hr) whereas, BAL macrophage numbers had significantly decreased from baseline (94.4 ± 3.1%) to (31.6 ± 21.2 % at 24 Hr and 64.2 ± 20.1% at 72 Hr) post challenge. In addition to increased inflammation, we also observed a significant increase in BAL cell gene expression of MMP-9 and significant increases in sCD14 and IL-8 from concentrated BALF 24 Hr post challenge. Numbers of infiltrating cells and other inflammatory parameters returned to baseline levels by 168 Hr following LPS challenge. In some studies, intramuscular injections of dexamethasone (Dex [1 mg/kg]) were administered 24 Hr prior and just prior to LPS challenge. Dex did not significantly reduce total BAL inflammation or IL-8 protein, but did significantly reduce percent neutrophils and sCD14 protein within the BALF 24 hours post challenge. Dex also significantly increased percent macrophages 24 Hr post challenge. These data show that segmental challenge with LPS produces an acute pulmonary neutrophilic inflammation, which is reduced by pretreatment with Dex. This reproducible, short-term, large animal model may help us to better understand neutrophillic inflammation pathways contributing to lung injury and host defense.

